# Case Report: A Rare Abdominopelvic Arteriovenous Malformation: Originating From Splenic Artery and Draining Into Portal Vein

**DOI:** 10.3389/fcvm.2022.916096

**Published:** 2022-06-23

**Authors:** Xin Li, Jiehua Li, Mo Wang, Junwei Wang, Lunchang Wang, Hao He, Ming Li, Quanming Li, Chang Shu

**Affiliations:** ^1^Department of Vascular Surgery, The Second Xiangya Hospital, Central South University, Changsha, China; ^2^Center of Vascular Surgery, Chinese Academy of Medical Sciences and Peking Union Medical College, Fuwai Hospital, Beijing, China

**Keywords:** abdominopelvic arteriovenous malformation, vascular malformation, splenic artery, portal vein, surgical resection

## Abstract

**Background:**

Abdominopelvic arteriovenous malformation is an uncommon congenital vascular lesion, for which the diagnosis and treatment are usually difficult. Though embolization and sclerotherapy are the primary treatment strategies, traditional surgical resection remains a valuable option.

**Case Presentation:**

Herein, we present a 32-year-old female diagnosed with a massive abdominopelvic arteriovenous malformation that originates from the splenic artery and drains into the portal vein. The vascular lesion was evaluated with multiple imaging modalities and then surgically resected successfully. The patient was discharged post-operatively on day 6 and free of symptoms during the 12-month follow-up.

**Conclusion:**

To our knowledge, the presented abdominopelvic arteriovenous malformation is the first to be reported in the literature, with such a rare condition originating from the splenic artery and draining into the portal vein.

## Background

Abdominopelvic arteriovenous malformation (AVM) is an uncommon vascular lesion, for which the etiology is usually congenital, and the diagnosis and treatment are difficult. The AVM is characterized by abnormal connections between supplying arteries and draining veins (1). It may begin with an asymptomatic lesion, but often progresses over time and expands secondary to trauma or hormonal change, such as that during puberty or pregnancy (2). The symptoms range from mild discomfort to high-output cardiac failure or life-threatening hemorrhage (3). Though embolization and sclerotherapy are the primary treatment strategies, traditional surgical resection remains a valuable option (4–6).

Most pelvic AVMs are supplied by internal iliac arteries, inferior mesenteric artery, or the median sacral artery (7–9), and are often drained into iliac, pudendal, or obturator veins (10–12). However, for the current case we present here, the AVM rarely had a single feeding artery from the splenic artery and two draining veins into the portal vein, which, to our knowledge, has not been reported before. The patient was asymptomatic and physical examination revealed aberrant abdominal vascular bruits. The massive abdominopelvic AVM was evaluated with multiple imaging modalities and successfully treated with surgical resection.

## Case Presentation

**Figure 1:**
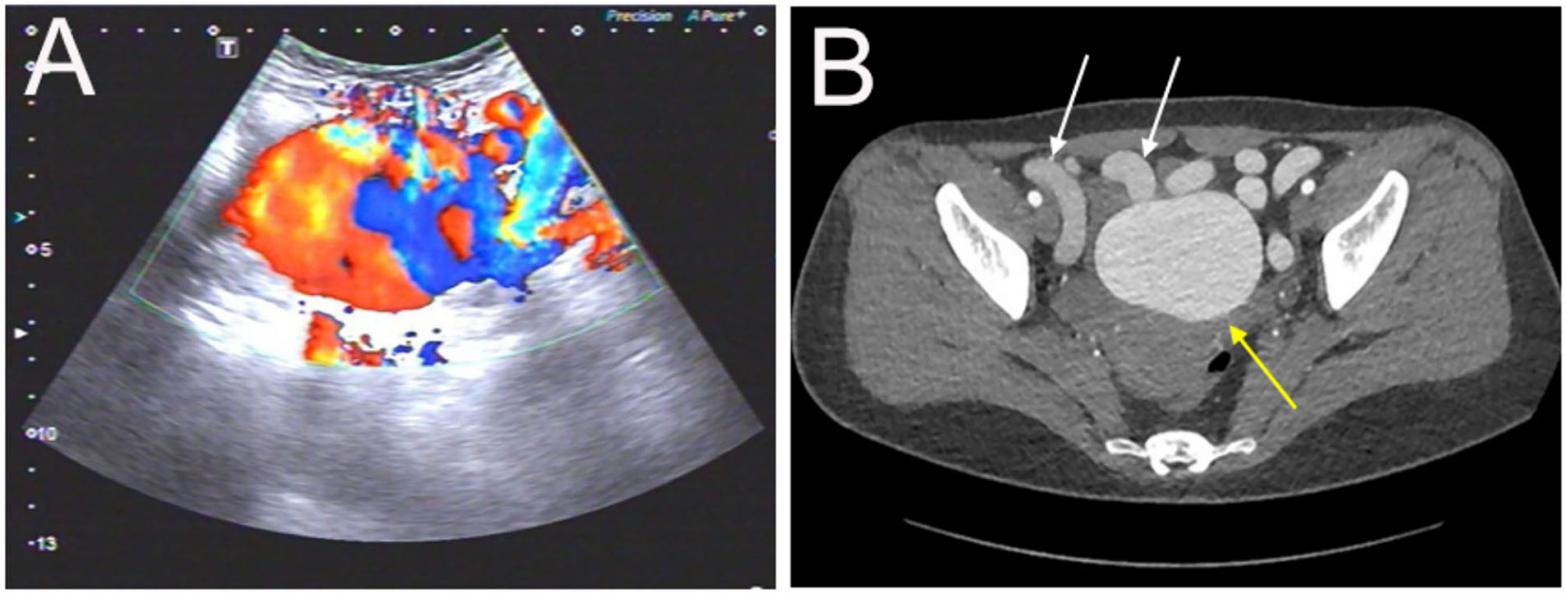
Evaluation of the abdominopelvic arteriovenous malformation (AVM) with color Doppler ultrasound and CT angiography. **(A)** The ultrasound revealed a pelvic mass with an abundant blood flow signal. **(B)** The axial view of CT angiography showed the nidus of AVM (yellow arrow) and dilated outflow veins (white arrows) (the aneurysm was measured as 72 ×56 mm).

**Figure 2:**
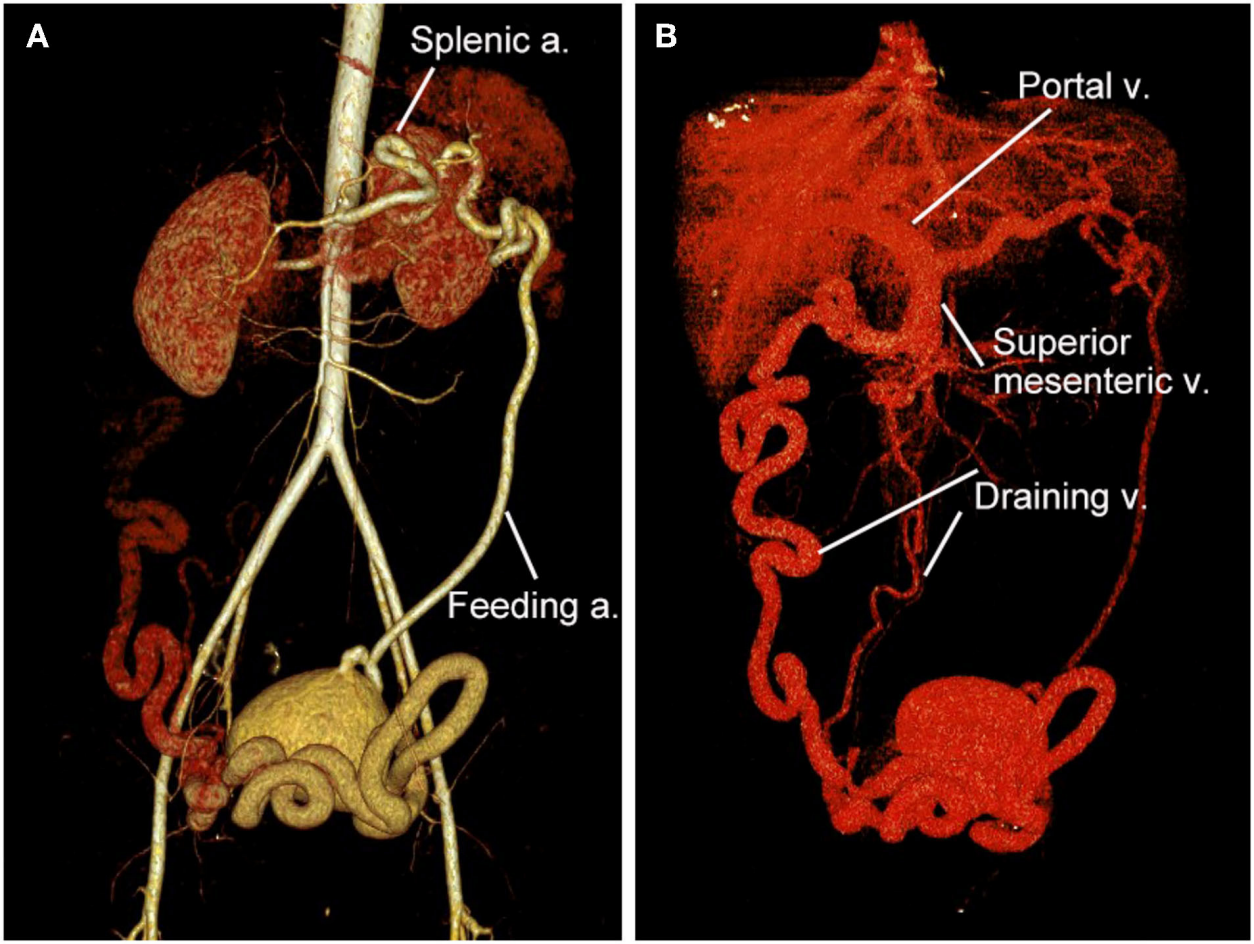
3D reconstruction of CT angiography of the abdominopelvic arteriovenous malformation (AVM). **(A)** Arterial phase of CT angiography showed the AVM had a single feeding artery originating from the splenic artery. **(B)** Venous phase of CT angiography showed the AVM had two draining veins into the superior mesenteric vein and then the portal vein.

**Figure 3:**
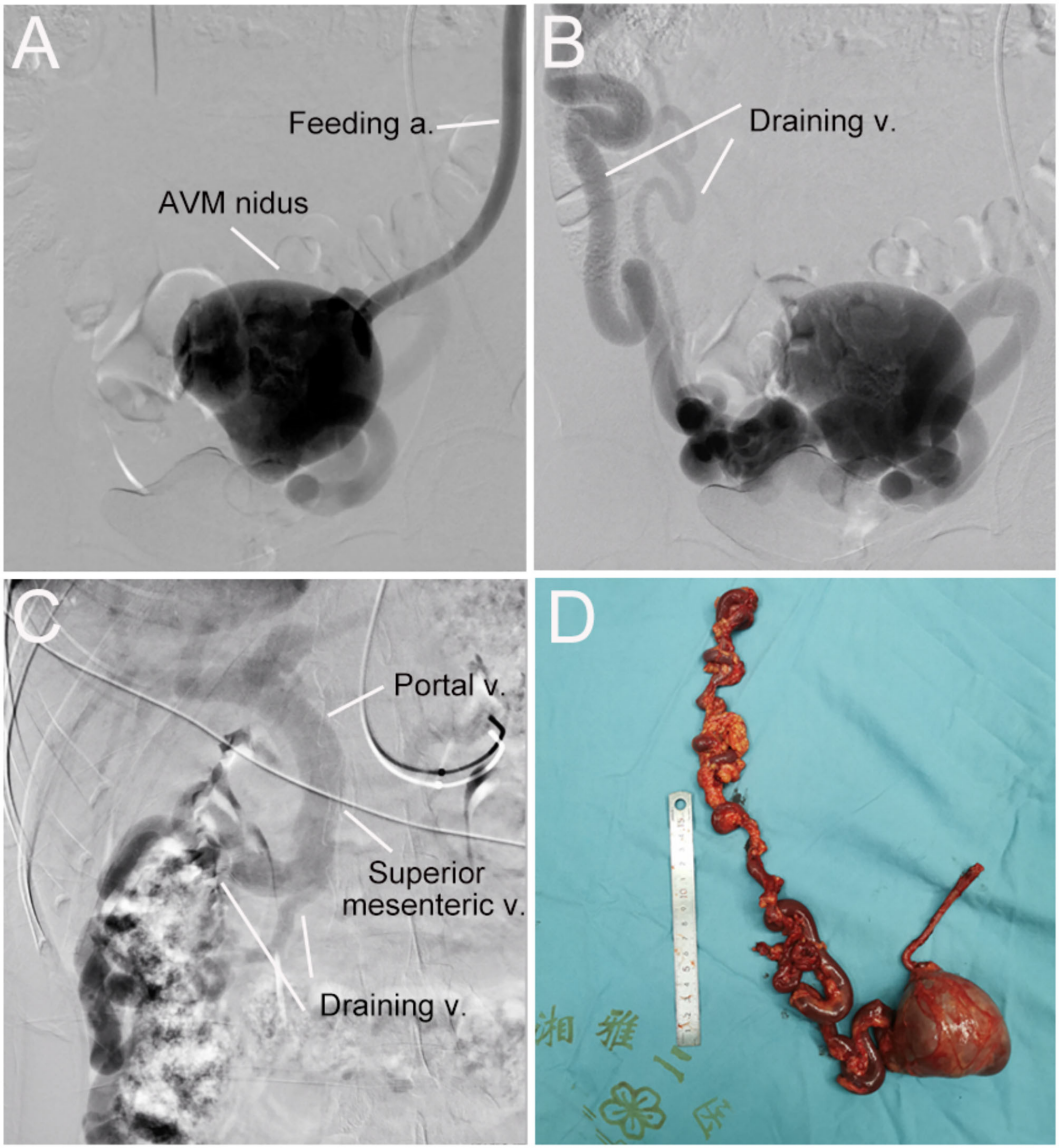
Digital subtraction angiography (DSA) and resected vascular lesion of the abdominopelvic arteriovenous malformation (AVM). **(A)** DSA showed the feeding artery and AVM nidus. **(B)** DSA showed the AVM nidus and two draining veins. **(C)** DSA showed draining veins flowed into the superior mesenteric vein and then the portal vein. **(D)** the AVM lesion was surgically removed successfully.

**Figure 4:**
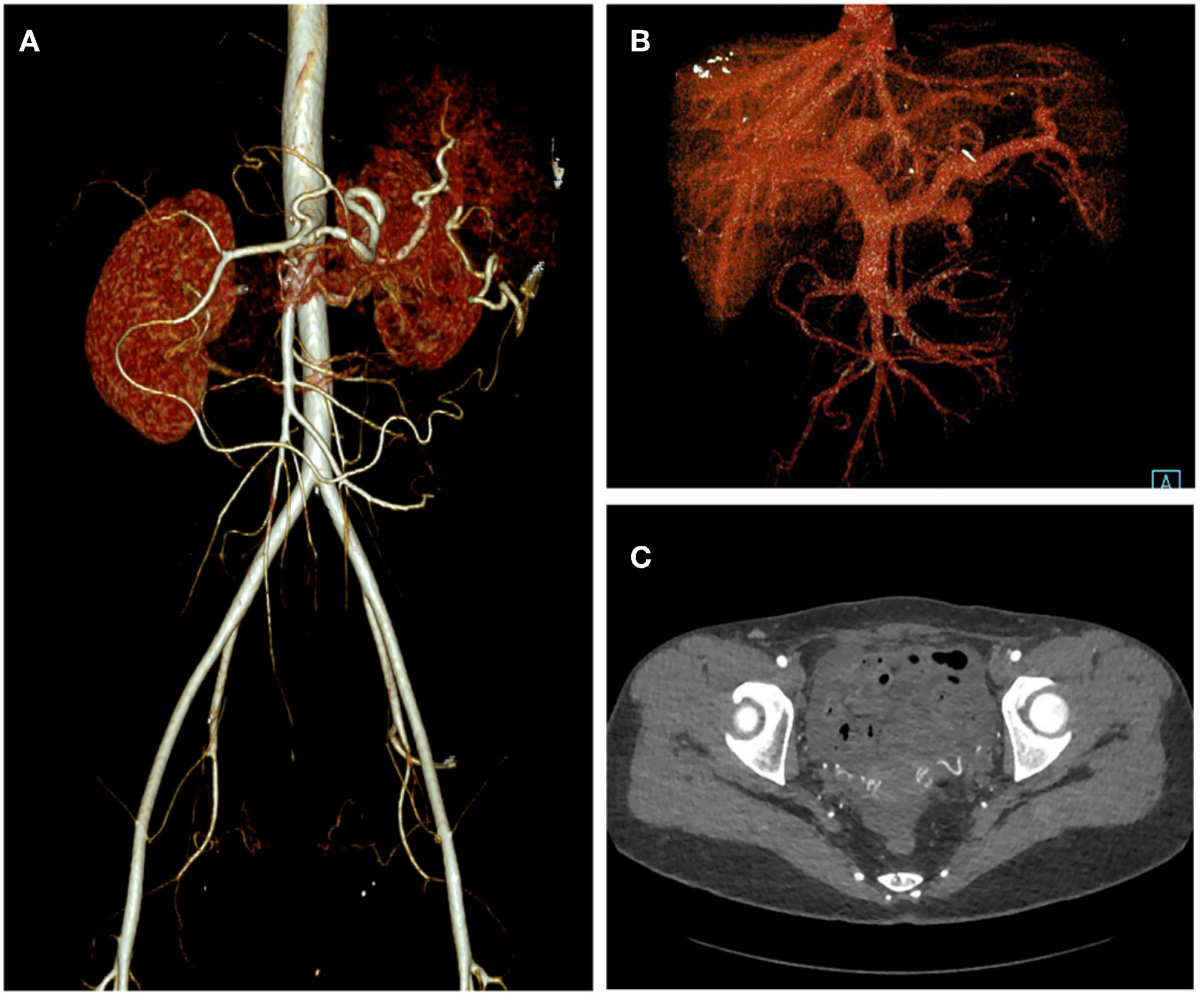
One-month post-operative CT angiography of the patient. Arterial phase **(A)**, Venous phase **(B)**, and axial view **(C)** of the CT angiography showed elimination of the abdominopelvic AVM.

A 32-year-old woman was admitted to our center due to the finding of a pelvic mass with hypervascularity 2 years ago. She was generally asymptomatic and had no related family history. On physical examination, her mass was not palpable, but vascular bruits could be easily auscultated on the abdomen, especially in the peri-umbilicus area. The ultrasound revealed a pelvic mass with an abundant blood flow signal (Figure 1A). CT angiography (CTA) showed the nidus of pelvic arteriovenous malformation (AVM) and aneurysmal-dilated vessel (measured 72 × 56 mm) (Figure 1B). The 3D reconstruction of CTA demonstrated that the AVM had a single feeding artery originating from the splenic artery and two draining veins into the superior mesenteric vein, and then, a portal vein (Figure 2). Digital subtraction angiography (DSA) was used to further evaluate the AVM (Figures 3A–C). It was confirmed that the AVM finally drained into the portal vein and there were no other feeding arteries from bilateral internal iliac arteries (Supplemenatry Video 1). Considering the risk of aneurysm rupture and hemorrhage, the patient was treated with surgical resection of the AVM and ligation of the feeding artery and draining veins (Figure 3D). She recovered well after the operation and was discharged post-operatively on day 6. The 1-month follow-up CTA showed elimination of the abdominopelvic AVM (Figure 4) and the patient was doing well during the following 12 months.

## Discussion And Conclusion

Arteriovenous malformation (AVM) belongs to the entity of vascular malformations, which are generally classified into slow-flow (venous, capillary, and lymphatic malformation) and fast-flow malformations (arteriovenous fistula and arteriovenous malformation) (13). AVM is an uncommon medical condition and accounts for 8% of vascular malformations (14). It could appear on almost any part of the body, including the abdominal and pelvic cavity (15). The composition of a typical AVM includes feeding arteries, draining veins, and the vascular shunting bypassing the capillary bed (also called “the nidus”) (3). Specifically for our case, the AVM had a feeding artery from the splenic artery, two draining veins into the portal vein, and significant dilated vascular “nidus” formed in the pelvic cavity.

Pelvic AVM may be asymptomatic in some patients, and present as a pulsatile or vascularized mass on pelvic imaging. However, AVMs often progress throughout life, and a variety of symptoms could occur, such as urinary frequency or dysuria, pelvic pain, life-threatening hemorrhage, and high-output cardiac failure. It is well-known that the growth of AVM could be stimulated by various factors, such as puberty, pregnancy, and trauma. According to the Schobinger classification system, there are typically four stages for AVM lesions: stage I (quiescent), stage II (expansive), stage III (destructive), and stage IV (cardiac decompensation) (16). The current case had a stage II pelvic AVM that was asymptomatic, but with a significantly dilated vascular lesion. She presented no signs of heart failure or portal hypertension.

The pelvic AVMs are usually first detected and evaluated by color Doppler ultrasound, which could offer an impressive overview of the lesion with hypervascularity signals (17). MR angiography and CT angiography are important diagnostic tools to visualize the feeding arteries, draining veins, and location of shunting, as well as flow dynamics (16). However, digital subtraction angiography (DSA) remains a pre-requisite for the treatment of AVMs. DSA *via* transarterial, transvenous, and sometimes direct percutaneous access could profoundly evaluate the AVM. The successful treatment of AVM depends largely on the diagnostic imaging, which needs to provide information on the location, extension, composition, vessel diameters, and hemodynamics of AVM.

Treatment of AVM is notoriously challenging, as inappropriate treatment could stimulate the AVM lesion into an aggressive growth state (18). Although an asymptomatic lesion can be observed, it is preferable to treat the AVM before it progresses. Surgical resection of AVM lesions is an effective strategy and has been regarded as the gold standard for a long period, but it carries the risk of massive bleeding, incomplete resection of the lesion, and injury to adjacent organs. In recent decades, endovascular therapy using various embolic and sclerosing materials, either alone or in a combination with surgical resection, has become a widely accepted treatment option for patients with AVMs (19, 20). However, the endovascular strategy could still lead to significant recurrence and complication rate (21). Thus, the optimal treatment of AVM should be individualized and be based on the certain condition of AVM and the experience of surgeons.

Here we presented a condition of AVM that might not have been described in the literature before, which had a massive vascular nidus in the pelvic cavity with feeding artery from splenic artery and draining veins into portal vein. The AVM was profoundly evaluated with color Doppler ultrasound, CT angiography, and catheter angiography, and it was demonstrated as a well-localized lesion that did not involve other organs or the pelvic wall. For this case, surgical resection was considered to be a superior strategy to endovascular therapy. As the AVM had relatively simple angioarchitectures without involving vital adjacent vital organs, surgical resection could provide a definite and effective treatment, while interventional therapy had the risk of incomplete or ectopic embolization.

The patient was treated successfully with surgical resection of the AVM and ligation of the feeding artery and draining veins. A major concern of the surgery was that removal of the AVM might significantly decrease the blood flow into the liver and cause ischemic liver injury. Fortunately, the patient recovered well and showed no signs of liver injury after the operation. The post-operative CTA showed no evidence of residual arteriovenous connections, and she lived uneventfully during the 12-month follow-up.

## Data Availability Statement

The original contributions presented in the study are included in the article/Supplementary Material, further inquiries can be directed to the corresponding author/s.

## Ethics Statement

The studies involving human participants were reviewed and approved by the institutional Ethics boards of the Second Xiangya Hospital. The patients/participants provided their written informed consent to participate in this study.

## Author Contributions

XL and JL drafted the manuscript. CS and QL designed the study. HH and ML revised the manuscript. MW, JW, and LW were responsible for the collection of data or analysis. All authors read and approved the final manuscript.

## Funding

This work was supported by the National Natural Science Foundation of China (grant numbers: 81870345 and 82120108005) and the Natural Science Foundation of Hunan Province, China (grant number: 2020JJ2054).

## Conflict of Interest

The authors declare that the research was conducted in the absence of any commercial or financial relationships that could be construed as a potential conflict of interest.

## Publisher's Note

All claims expressed in this article are solely those of the authors and do not necessarily represent those of their affiliated organizations, or those of the publisher, the editors and the reviewers. Any product that may be evaluated in this article, or claim that may be made by its manufacturer, is not guaranteed or endorsed by the publisher.

## Supplementary Material

Supplementary Video Digital subtraction angiography of the massive abdominopelvic arteriovenous malformation.
